# Cell death induced by mycotoxin fumonisin B_1_ is accompanied by oxidative stress and transcriptional modulation in *Arabidopsis* cell culture

**DOI:** 10.1007/s00299-022-02888-5

**Published:** 2022-06-25

**Authors:** Alessandra Lanubile, Roberto De Michele, Martina Loi, Safieh Fakhari, Adriano Marocco, Costantino Paciolla

**Affiliations:** 1 Department of Sustainable Crop Production, Università Cattolica del Sacro Cuore, via Emilia Parmense 84, 29122 Piacenza, Italy; 2 Institute of Biosciences and Bioresources, National Research Council of Italy, corso Calatafimi 414, 90129 Palermo, Italy; 3 Institute of Sciences of Food Production, National Research Council of Italy, via Amendola 122/0, 70126 Bari, Italy; 4 Department of Biology, Università degli Studi di Bari Aldo Moro, via E. Orabona 4, 70125 Bari, Italy

**Keywords:** Fumonisins, *Arabidopsis*, Antioxidant enzymes, Gene expression, Cell death, Reactive species

## Abstract

**Key message:**

**Fumonisin B**_**1**_ **induces rapid programmed cell death in Arabidopsis cells, oxidative and nitrosative bursts, and differentially modulates cell death responsive genes. Glutathione is the main antioxidant involved in the stress response.**

**Abstract:**

Fumonisin B_1_ (FB_1_) is a fungal toxin produced by *Fusarium* spp. able to exert pleiotropic toxicity in plants. FB_1_ is known to be a strong inducer of the programmed cell death (PCD); however, the exact mechanism underling the plant–toxin interactions and the molecular events that lead to PCD are still unclear. Therefore, in this work, we provided a comprehensive investigation of the response of the model organism *Arabidopsis thaliana* at the nuclear, transcriptional, and biochemical level after the treatment with FB_1_ at two different concentrations, namely 1 and 5 µM during a time-course of 96 h. FB_1_ induced oxidative and nitrosative bursts and a rapid cell death in *Arabidopsis* cell cultures, which resembled a HR-like PCD event. Different genes involved in the regulation of PCD, antioxidant metabolism, photosynthesis, pathogenesis, and sugar transport were upregulated, especially during the late treatment time and with higher FB_1_ concentration. Among the antioxidant enzymes and compounds studied, only glutathione appeared to be highly induced in both treatments, suggesting that it might be an important stress molecule induced during FB_1_ exposure. Collectively, these findings highlight the complexity of the signaling network of *A. thaliana* and provide information for the understanding of the physiological, molecular, and biochemical responses to counteract FB_1_-induced toxicity.

**Supplementary Information:**

The online version contains supplementary material available at 10.1007/s00299-022-02888-5.

## Introduction

Fumonisins are toxic metabolites produced by phytophatogenic fungi belonging to the *Fusarium* species. *Fusarium proliferatum*, *F. verticillioides*, *F*. *oxysporum*, and *F*. *fujikuroi* are among the main producers, responsible for the contamination of several crops worldwide (Braun and Wink 2018). *Fusarium* spp. are able to contaminate several crops of agronomic and economic relevance, such as tomato, maize, rice, and sunflower, hence representing an important health and economic concern globally (Khodaei et al. 2021). *Fusarium* spp. can infect plants at different stages of their development, and the production of fumonisins can be an important pathogenicity and virulence factor. Fumonisin B_1_ (FB_1_) is the most toxic and prevalent one (Cendoya et al. 2018), and it is classified in group 2B by the International Agency of Research on Cancer (IARC 1993), resulting as a possible human carcinogen. Besides, it displays pleiotropic toxicities in animals (neurotoxicity, hepatotoxicity, and nephrotoxicity) and plants (chlorosis, necrosis, wilting, reduced growth and seed germination, and death) (Renaud et al. 2021).

Fumonisins are polyketides composed by an aminopolyol backbone structure with two tricarballylic acid side chains and an amine moiety. Their toxicity can be ascribed to their chemical structure, which resembles sphinganine, a precursor of cell membrane sphingolipids. In particular, the tricarballylic acid side chains and the amine moiety are the main toxic determinants (Renaud et al. 2021). At cellular level, fumonisins act as inhibitor of the isoenzyme LONGEVITY ASSURANCE GENE ONE HOMOLOG1 (LOH1), leading to the accumulation of specific long chain bases (LCB) that induce a SA-dependent cell death response (Luttegeharm et al. 2015, 2016). The imbalance in LCB, together with the induction of oxidative stress at cytoplasm and ER level, triggers different downstream signaling pathways, eventually leading to the programmed cell death (PCD) in plants (Qin et al. 2017; Iqbal et al. 2021). Conversely, the overexpression of LOH1 did not disclose any resistance to the mycotoxin (Luttegeharm et al. 2015, 2016). Furthermore, LOH1 inhibition induced the accumulation of specific LCB sphingolipids leading to a salicylic acid (SA)-mediated PCD (König et al. 2021).

FB_1_ can also elicit a rapid PCD, known as hypersensitive response (HR), usually initiated by a pathogen attack and limited to the cells, which are in direct contact with the pathogen (Salguero-Linares and Coll 2019). This response includes chromatin condensation, phenols and callose deposition, phytoalexin accumulation, rapid accumulation of reactive oxygen species (ROS) and reactive nitrogen species (RNS), and the expression of pathogenesis-related (PR) proteins (Zhang et al. 2015).

Oxidative stress also plays a pivotal role in determining FB_1_ toxicity. After FB_1_ exposure, increased levels of ROS, lipid peroxidation and oxidative DNA damage can be observed in vitro and in vivo due to an impairment of the redox homeostasis (Liu et al. 2019). However, the exact mechanism behind the antioxidant defense system and crosstalk among the phytohormones, sphingolipids ratio, and the resulting responses at nuclear and organelle level that led to PCD is still unclear. Likewise, it remains to be established whether the oxidative stress is caused by FB_1_ or a consequence of other events that take place upon exposure (Zeng et al. 2020; Iqbal et al. 2021).

*Arabidopsis thaliana* is a model organism, which has been extensively used to study gene expression and toxicity mechanisms induced by FB_1_ exposure at cellular level (Iqbal et al. 2021). Understanding the molecular and biochemical pathways induced by this mycotoxin is essential to counteract the toxic effects of fungal and mycotoxin contamination, and possibly to identify bioactive compounds able to minimize or neutralize them or key components that confer resistance to mycotoxin or fungal contamination. Indeed, many bioactive compounds are able to regulate these signaling pathways and to counteract the mycotoxin-induced oxidative stress (Loi et al. 2020a), and antioxidant systems to actively participate in the defense system against fungi and their mycotoxins (Loi et al. 2020b; Maschietto et al. 2016; Lanubile et al. 2015).

Therefore, the aim of this work was to provide a comprehensive investigation of *A. thaliana* responses upon FB_1_ exposure at the nuclear, transcriptional, and biochemical level, with particular attention to the nuclear morphology, the role of antioxidant components, and the key genes associated to cell death, to shed light on the stress response induced by FB_1_ in *Arabidopsis* cell cultures.

## Materials and methods

### Cell cultures and treatments

The *Arabidopsis* cell line derived from hypocotyls were dissected from young plantlets of Arabidopsis thaliana (L) Heynh. Ecotype Landsberg erecta (Ler), and subcultured in liquid AT3 medium (Carimi et al. 2005). For subculture cycles, 2 mL of packed cell volume was placed in 250 mL Erlenmeyer flasks containing 50 mL of liquid medium. Cells were subcultured in fresh medium at 7 days intervals and maintained in a climate chamber on a horizontal rotary shaker (80 rpm) at 25 °C with a 16 h light/8 h dark photoperiod and a light intensity of 70 µmol m^−2^ s^−1^. Treatments were performed on 3-days-old cultures. FB_1_ (AppliChem, Germany) was dissolved in dimethyl sulfoxide (DMSO) at a 10 mM stock concentration. Flasks were treated with 5 µL (1 µM FB_1_) or 25 µL (5 µM FB_1_). Control flasks were mock treated with 25 µL DMSO, unless otherwise specified. Cells were analysed and collected 1, 3, 6, 24, 48, 72 and 96 h after treatment. Cell growth was estimated by measured the fresh weight of the cultures and vacuum filtered on a filter paper.

### Cell death assessment and analysis of nuclear morphology

Cell death was estimated by Evan’s blue staining method. Evan’s blue is a dye that only stains dead cells. Briefly, 2 mL cell cultures were stained in a tube by adding 50 µL of a 0.5% w/v Evan’s blue (Sigma-Aldrich) solution. After 15 min incubation, cells were filtered through a chromatographic column (Bio-Rad, USA) and washed three times with distilled water to remove excess dye. Columns were capped and filled with 2 mL elution solution (50% v/v methanol, 1% w/v sodium dodecyl sulfate) and incubated 30 min at 55 °C to promote dye release. Once filtered, the A600 of the solution was read at a spectrophotometer (Biotek, USA). To compensate for cell number, due to different time of culture or growth inhibition caused by treatments, the same procedure was applied to 2 mL of culture boiled for 7 min, whose A600 value was then set as 100% dead cells.

Nuclei were stained and visualized with a fluorescence microscope (DMR, Leica) as reported in De Michele et al. (2009).

### ROS and RNS quantification

ROS and RNS were quantified by fluorimetric analysis using specific fluorescent dyes, as in Sharaf et al. (2019). In particular, 2,7-dichlorodihydrofluorescein diacetate (H_2_DCF-DA) is an intracellular marker that measures the level of oxidation inside a cell (Chen et al. 2010a, b); dihydroethidium (DHE) is a specific marker for the superoxide anion (O_2_^–^) (Nazarewicz et al. 2013); 4-Amino-5-methylamino-2’,7’-difluorofluorescein diacetate (DAF-FM-DA) is an intracellular specific marker for nitric oxide (NO) (Kojima et al. 1999); Aminophenyl fluorescein (APF) is a marker for both peroxynitrite (ONOO^−^) and hydroxyl radical (⋅OH), since the dye is unable to discriminate between the two molecules (Setsukinai et al. 2003). All dyes were from Cayman Chemicals, USA, and were dissolved in DMSO. Two mL of culture was deposited in a well in a transparent 12-well polypropylene plate (Greiner, Germany) and brought to pH 7.5 by adding 20 µL 10 mM Tris buffer (final concentration 100 µM). Then, 2 µL of dye was added to the culture, with final dye concentration of 10 µM (H_2_DCF-DA, DHE, APF) or 5 µM (DAF-FM-DA). Plates were incubated at 25 °C for 30 min on agitation (100 rpm) in the dark. Fluorescence was measured using a Synergy H1 reader (Biotek, USA) with a bottom reader mode and gain set to 80 (H_2_DCF-DA and APF) or 100 (DHE and DAF-FM-DA) and bandwidth of 9 nm. To avoid the formation of cell clumps, which affect the homogeneity of the fluorescence readout, the measurement was made in 21 different points on the well surface and averaged (“area scan” mode). Excitation and emission wavelengths for each dye were 495/525 Ex/Em for H_2_DCF-DA; 495/515 Ex/Em for DAF-FM-DA and APF; for DHE, we used 405/570 nm Ex/Em instead of the commonly used 480/580 nm Ex/Em because the former setup was proved to be more selective in detecting·O_2_^–^, rather than the unspecific oxidized byproduct 2-OH-ethidium.

Extracellular H_2_O_2_ was measured by using the xylenol orange method. Briefly, 2 mL of culture was filtered through a chromatographic column (Poly-Prep; Bio-Rad, USA) to separate cells from the growth medium. An aliquot of 500 mL of the flow through was added to an equal volume of assay reagent (500 mM ferrous ammonium sulfate, 50 mM H_2_SO_4_, 200 mM xylenol orange, and 200 mM sorbitol) and incubated for 45 min in the dark. The H_2_O_2_-mediated oxidation of Fe^2+^ to Fe^3+^ was determined by measuring the A_560_ of the Fe^3+−^xylenol orange complex. All reactions were carried out at least in duplicate, and their reproducibility was checked.

Intracellular H_2_O_2_ was determined by fluorescence using dihydrorhodamine 123 (DHR123; Sigma-Aldrich, St. Louis, MO) (Qin et al. 2017). Briefly, an aliquot of frozen cell culture (0.5 g) was incubated for 15 min with 10 µL of a solution containing sucrose (30% w/v) and DHR123 115 µM. Green fluorescence related to intracellular H_2_O_2_ was observed in a fluorescent microscope (DMLS, Leica) with an excitation filter of 450–490 nm and a barrier filter of 510 nm.

### Gene expression and DNA analysis

Genomic DNA was extracted by grinding cells with liquid nitrogen, followed by the Doyle and Doyle method (Carimi et al. 2004) and quantified at a spectrophotometer. For DNA fragmentation analysis, 10 µg of each sample was electrophoresed on 1% (w/v) agarose gels containing 1 × TAE (40 mM Tris–acetate, 1 mM EDTA) and stained with ethidium bromide.

Expression of the senescence marker *SAG12* was performed as in (Carimi et al. 2004), by using 18S as an internal standard (Ambion, USA). Cells from 14 days old culture were used as positive control for *SAG12* expression (Carimi et al. 2004).

For real-time reverse transcription-PCR (RT-PCR) gene expression, *Arabidopsis* cells were ground under liquid nitrogen with a pestle and mortar, and total RNA extraction and purification were carried out based on Lanubile et al. (2013, 2015). Real-time experiments were performed on cells collected at 24 and 48 h after treatment with 1 and 5 µM FB_1_ solution using the FluoCycle™ II SYBR Green master mix (EuroClone S.p.a., Milan, Italy) and the CFX-96 device (Bio-Rad, Hercules, CA, U.S.). One µg of total RNA was used for cDNA synthesis using the High Capacity cDNA Reverse Transcription Kit (Thermo Fisher Scientific Inc. Waltham, Massachusetts, U.S.). Twenty ng of single strand cDNA determined by fluorometric assay (Qubit, Thermo Fisher Scientific) were used for real-time RT-PCR. Relative RT-PCR was performed under the following conditions: 95 °C for 3 min and 40 cycles at 95 °C 15 s, 55–60 °C for 30 s, followed by a melting curve analysis (Lanubile et al. 2013, 2015). Samples and template-free negative controls from each of three independent biological replicates were assayed in triplicate (technical replicates). Gene-specific primers are listed in Supplemental Table S1. Relative quantification was normalized to the reference housekeeping gene *Actin2*. Fold changes (FC) values in gene expression were calculated using the 2^−ΔΔCt^ method (Schmittgen and Livak 2008) and calibrated on the mock-treated cells.

### Determination of proteins extraction and quantification

One gram of cell culture was harvested by filtration as described above and ground with liquid nitrogen in a porcelain mortar. Then, the extraction buffer consisting of Tris–HCl 50 mM pH 7.8 0.05% w/v cysteine and 0.1% w/v bovine albumin was added in a ratio 1:2 w/v. The homogenate was centrifuged at 1000×*g* for 5 min. The supernatant was re-centrifuged at 25,000×*g* for 20 min and the resulting supernatant was desalted by dialysis against 50 mM Tris–HCl pH 7.8, and used for enzyme activity measurements and for the electrophoretic analyses. All procedures were carried out at 4 °C. The protein content was quantified with a Protein Assay kit from Bio-Rad (Hercules, CA, USA) with bovine serum albumin as the standard.

### Enzyme activity measurements

Enzyme activities were determined spectrophotometrically, by monitoring the rate of substrate oxidation or product formation at specific wavelengths. In particular, APX (EC 1.11.1.11), CAT (EC 1.11.1.6), POD (EC 1.11.1.7), SOD (EC 1.15.11), MDHAR (EC 1.6.5.4), and GR (EC 1.6.4.2) were tested according to Paciolla et al. (2008) and Mastropasqua et al. (2012). DHAR (EC 1.8.5.1) was determined according to Loi et al. (2020a, b).

### Ascorbate and glutathione pools

For ascorbate and glutathione determinations, 0.5 g cells were packed in 2 mL tubes, resuspended in 1.5 mL of a 5% w/v metaphosphoric acid solution and frozen in liquid nitrogen.

Ascorbate and glutathione pools were determined according to Loi et al. (2019).

### Measurement of oxidation level

The oxidation level of the cells was monitored by measuring the end product malondialdehyde (MDA), which indicates the level of lipid peroxidation and sugar and amino acid oxidation. Briefly, 0.7 g of *Arabidopsis* cells were grounded in a porcelain mortar with liquid nitrogen and dissolved with 0.1% trichloroacetic acid with a ratio of 1:4 (w/v). After centrifugation at 12,000×*g* for 10 min, the supernatant was diluted 1:1 with a solution containing 20% trichloroacetic acid and 0.5% thiobarbituric acid (TBA) and incubated for 30 min at 90 °C. The reaction was stopped in ice and the samples centrifuged at 12,000×*g* for 10 min. The resulting supernatant was used for the determination of MDA-TBA complex by spectrophotometric measurement at 532 nm (extinction coefficient 155 mM^−1^ cm^−1^). The obtained absorbance was corrected subtracting the value of unspecific turbidity at 600 nm.

### Electrophoretic analyses

#### Native-PAGE

Native-PAGE was performed on PAGE (7.5% T; 4.0% C). Fourty µg of total proteins were loaded in each lane. The electrophoresis was performed in a Mini Protean System (Bio-Rad, Segrate, Italy) filled with running buffer (25 mM Tris and 1.9 M glycine). The run was performed at 32 mA for 3 h. After the electrophoretic run, gels were used for activity staining for the different enzymes. Activity staining was performed by incubating the gels in specific buffers. APX, CAT, GR were detected as described by Paciolla et al. (2016), DHAR according to Loi et al. (2020a, b) while SOD by Villani et al. (2021). For POD activity staining, the gel was incubated in Tris–acetate 0.1 M, pH 5 containing H_2_O_2_ 0.32 mM and 1-methoxynaphthol 1 mM. After incubation at 27 °C for 15 min, POD appeared as blue bands on a light background.

#### Protein thiol labeling

Protein SH groups were labeled with the fluorescent probe monobromobimane (mBBr) according to procedure reported by Gobin et al. (1997). 150 µg of total proteins were loaded on sodium dodecyl sulphate (SDS) gel (10% T, 4% C). SDS-PAGE was performed according to Laemmli (1970). The protein content was assayed with the Bio-Rad kit. The electrophoretic run was performed as previously described for Native-PAGE. After the run, the proteins was fixed with trichloroacetic acid 12% (w/v) for 1 h and then the gel was incubated in a solution consisting of 40% methanol, 10% acetic acid, and 50% water for 10 h to remove excess mBBr. The fluorescence of thiol-bound mBBr was detected by placing the gel on a UV-transilluminator (365 nm). The resulting fluorescence emission is indicative of the thiol presence in the analyzed proteins (Paciolla et al. 2001). The intensity of fluorescent bands was analysed with UTHSCA Image Tool software.

### Statistical analyses

Three independent biological replicates were performed for all experiments. For cell growth, mortality, ROS and RNS analysis means and standard deviations are presented. Variance among replicates was first tested by F-test, to check for equal or unequal distribution. Then, treatments were compared for significant difference at *p* = 0.05 by *t* test.

For gene expression and other parameters analyzed, standard deviations of the means were calculated on three biological replicates. One-factor analysis of variance (ANOVA), followed by Tukey’s HSD test (*p* < 0.05), was performed on the observed means of FC gene expression other studied parameters values to set significant differences between times of treatment (24 and 48 h for gene expression; 24, 48 and 72 h for enzyme activity, ascorbate, glutathione and lipid peroxidation) within each FB_1_ concentration and between FB_1_ concentrations (1 and 5 µM) within each time of treatment.

## Results and discussion

### Fumonisin B_1_ induces rapid cell death in ***Arabidopsis*** cell cultures

Previously, we have shown that *Arabidopsis* cell culture is a good model for studying natural senescence and induced programmed cell death (PCD), namely by high concentration of cytokinins (Carimi et al. 2004, 2005) and heavy metals (De Michele et al. 2009). Under normal subculturing conditions, cells experience an exponential growth phase for the first 10 days, followed by a stationary lag phase and an eventual decline due to starvation (Carimi et al. 2005). To study the effect of FB_1_ in *Arabidopsis* cell cultures, we treated cells at the beginning of their linear growth phase, before they started to senesce.

**Fig. 1:**
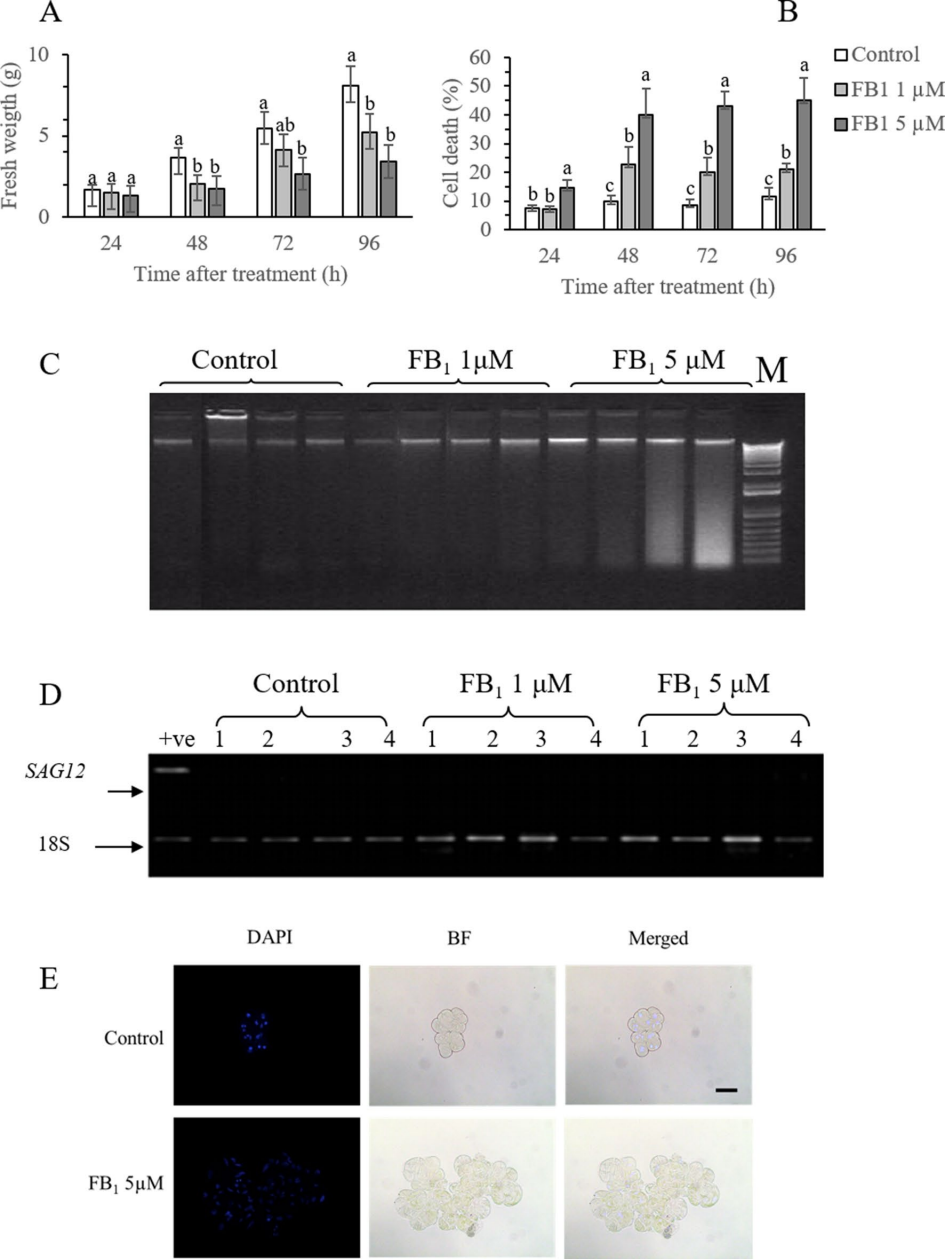
Characterization of FB_1_ toxicity. **A** Growth of cell cultures mock-treated with DMSO (Control) or treated with 1 or 5 µM FB_1_; **B** cell mortality of cultures mock-treated with DMSO (Control) or treated with 1 or 5 µM FB_1_; **C** DNA integrity, as assessed by agarose gel electrophoresis. *M* = 1 kb plus marker; **D** *SAG12* expression analysis by RT-PCR; **E** Nuclei condensation as assessed by DAPI staining, bright field (BF) and merged images. Bar = 50 µm Vertical bars indicate ± sd. Letters indicate significantly different samples at each time point, according to *t* test with *p* < 0.05. Experiments refer to three independent biological replicates. Panels **C**, **D** are representative images of three independent measurements

*Arabidopsis* cells suspension cultures were treated with two different FB_1_ concentrations, 1 and 5 µM. Mock-treated control cells maintained a linear growth pattern as assessed by fresh weight measurements, doubling between one and three days from treatment (Fig. 1A). Cells treated with FB_1_ showed a marked reduction in growth, which was more severe in the 5 µM treatment. Four days after treatment, cells with 5 µM FB_1_ weighted less than half of controls. To determine whether the impairment in growth was an induction of lag phase or rather depended on increased mortality, we quantified dead cells. Whereas control cells showed a physiological 10% rate of dead cells along all the experiments, as expected from their growth curve (Carimi et al. 2005), cells treated with FB_1_ dramatically increased their mortality (Fig. 1B). Cell death increased as early as 1 day after treatment with 5 µM FB_1_, and eventually reached 45%.

In plants, cell death may be characterized by a wide range of features, from necrosis to full PCD. A typical marker of PCD, especially the “slow” events such as natural and induced senescence, is the gradual condensation of DNA within the nuclei, often appearing with a sickle shape, as opposed to the relaxed and round aspect of healthy nuclei. The following event during PCD is the cleavage of DNA in the inter-histonic spaces, leading to a ladder band pattern after electrophoresis (Carimi et al. 2005; De Michele et al. 2009). Conversely, necrosis or “fast” PCD events such as the HR often present a chaotic degradation of the DNA molecules, resulting in a smear after electrophoresis. To determine whether FB_1_-induced cell death showed typical PCD hallmarks, we checked DNA integrity by looking at its fragmentation pattern and nuclear condensation. When run in a gel, DNA from control cells was intact, as indicated by the high molecular weight band (Fig. 1C). Conversely, treatment with 5 µM FB_1_ resulted in an eventual DNA degradation in a smear, in agreement with the rapid and potent toxic effect observed in cell death measurements. As a further test for characterizing FB_1_-induced cell death, we analyzed the expression of *SAG12*, a well-known specific marker for senescence, induced during both natural and induced senescence in *Arabidopsis* cell cultures (Carimi et al. 2005; De Michele et al. 2009). FB_1_-treated cells, as well as healthy control cells, never showed *SAG12* induction (Fig. 1D), suggesting that the cell death event did not resemble an accelerated senescence, thus differing from other PCD inducers such as BAP and cadmium (Carimi et al. 2005; De Michele et al. 2009). On the other hand, several nuclei of FB_1_-treated cells showed sickled condensed nuclei when looked at the microscope, as opposed to control cells (Fig. 1E). Nuclear condensation is present in rapid PCD processes such as the HR triggered by pathogens. Since FB_1_ is a mycotoxin produced by a plant pathogen, it is likely that the cell death caused by FB_1_ treatment resembles a HR-like PCD event. In agreement with our observation, Asai and colleagues already had observed typical PCD markers such as positive TUNEL nuclei in *Arabidopsis* protoplasts treated with FB_1_ (Asai et al. 2012).

### Fumonisin B_1_ induces an oxidative and nitrosative burst

It is well known that in plants the HR response caused by an incompatible pathogen interaction is characterized by an early oxidative and nitrosative burst (Romero-puertas et al. 2004). In particular, hydrogen peroxide (H_2_O_2_) and nitric oxide (NO) are two players identified first in HR. Yet, the chemistry and the crosstalk among the different members of reactive oxygen and nitrogen species (ROS and RNS) is complex, and may differ greatly depending on the concentration, timing and localization of each molecule. ROS comprise the above-mentioned H_2_O_2_ but also the superoxide anion (O_2_^−^), hydroxyl radicals (OH) and singlet oxygen (_1_O^2^), produced during electron transport chains in chloroplasts and mitochondria, or by oxidases and peroxidases in peroxisomes and in the apoplast. RNS, besides the well-known NO, include the peroxynitrite anion (ONOO^−^), which forms by reaction of NO with O_2_^−^.

**Fig. 2:**
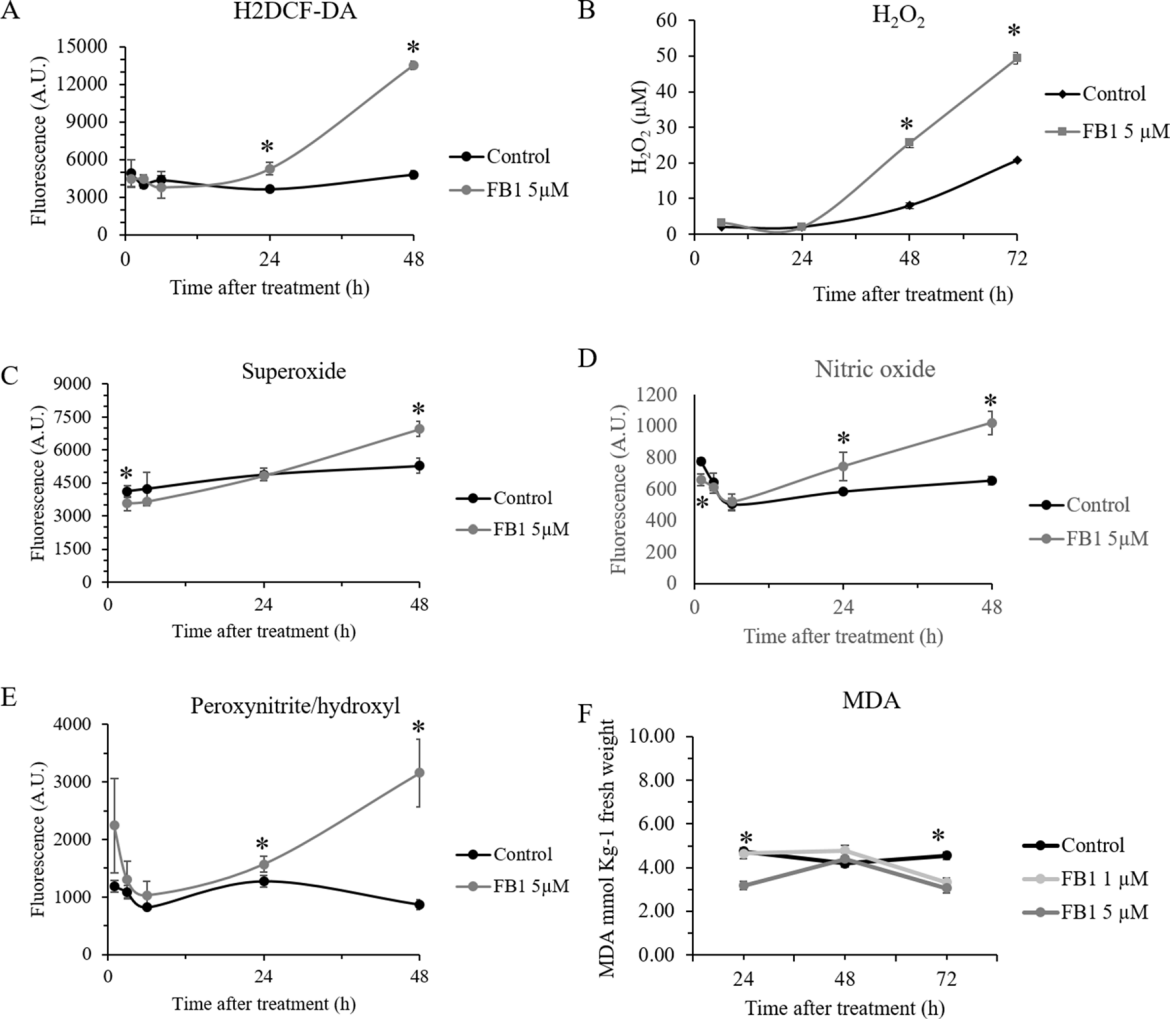
ROS and RNS production in mock-treated and 5 µM FB_1_ treated cells. **A** Intracellular oxydating events, as assessed by H_2_DCF-DA fluorescence; **B** extracellular H_2_O_2_; **C** superoxide anion O_2_^−^; **D** nitric oxide NO; **E** peroxynitrite/hydroxyl radical ONOO^−^/OH; **F** lipid peroxidation, as assessed by MDA content. Vertical bas indicate ± sd. Asterisks indicate significantly different samples at each time point, according to *t* test with *p* < 0.05. Experiments refer to three independent biological replicates

To add on the complexity of the crosstalk among these players, it is known that NO and H_2_O_2_ can interact to promote the formation of^.^OH and _1_O^2^, but NO can also scavenge H_2_O_2_, thus protecting plant cells from damage. To assess whether FB_1_ treatment, by mimicking an HR response, caused and oxidative and/or nitrosative burst, we measured ROS and RNS production over time. Since ROS and RNS can act as signaling molecules, as well as late downstream cell death effectors, we extended our analysis as early as 1 h after treatment, focusing with 5 µM FB_1_ concentration, which gave the strongest response in the cell physiology assays. As a generic measure of oxidative stress, the levels of the fluorescence dye H_2_DCF-DA maintained at the same level of control cells for the first six hours. At 24 h, and even more at 48 h after treatment, cells experienced a high level of oxidative stress (Fig. 2A). Looking at the specific reactive species involved, we observed that the extracellular H_2_O_2_ release, as well as intracellular O_2_^−^ levels, were late events, being significantly higher than control after only one day of treatment (Fig. 2B and C). Conversely, NO and ONOO^−^/OH increased as early as 24 h after FB_1_ exposure (Fig. 2D and E). Being ONOO^−^ produced as result of reaction between NO and O_2_^−^, it comes with no surprise that its pattern followed those of the parent species. It is interesting to notice that an early NO production, preceding H_2_O_2_, was similarly observed in *Arabidopsis* cell cultures treated with the heavy metal cadmium, and it was causally linked to the onset of programmed cell death (De Michele et al. 2009). It is tempting to speculate that the concomitant presence of NO, H_2_O_2_, and possibly other ROS and RNS species, is therefore a general feature of programmed cell death in plants.

In addition, intracellular H_2_O_2_ was evaluated. Its level was significantly higher than the control for both treatments during all the assay, with 1 µM FB_1_ having the utmost effect (data not shown).

We then assessed the level of the oxidative damage by MDA assay. A significant higher level of MDA content at 6 h of the treatment was observed indicating increased oxidative status. An unexpected significantly lower MDA level was found at 24 h and 72 h for 5 µM FB_1_ and at 72 h for 1 µM FB_1_, as compared to control cells (Fig. 2F).

A possible explanation could be higher GSH level found in the FB_1_ treated-cells when compared with control (see “Antioxidant compounds and enzymes involved in the ascorbate–glutathione cycle”); GSH can prevent damage to important cellular components as membranes caused by reactive oxygen species. It is able to reach directly, free radicals, peroxides, lipid peroxides, and heavy metals and is involved in pathogen resistance (Noctor and Foyer 1998). Indeed, GSH differs from other metabolites that may play a similar role because of the presence of specific enzymes that link GSH with H_2_O_2_ metabolism, the stability of the corresponding oxidized form, and the ability to be recycled to reduced form through a powerful enzymatic system that depends on the electron transport molecule NAD(P)H (Foyer and Noctor 2011).

### Differential modulation of cell death responsive genes during FB_1_ exposure

**Fig. 3:**
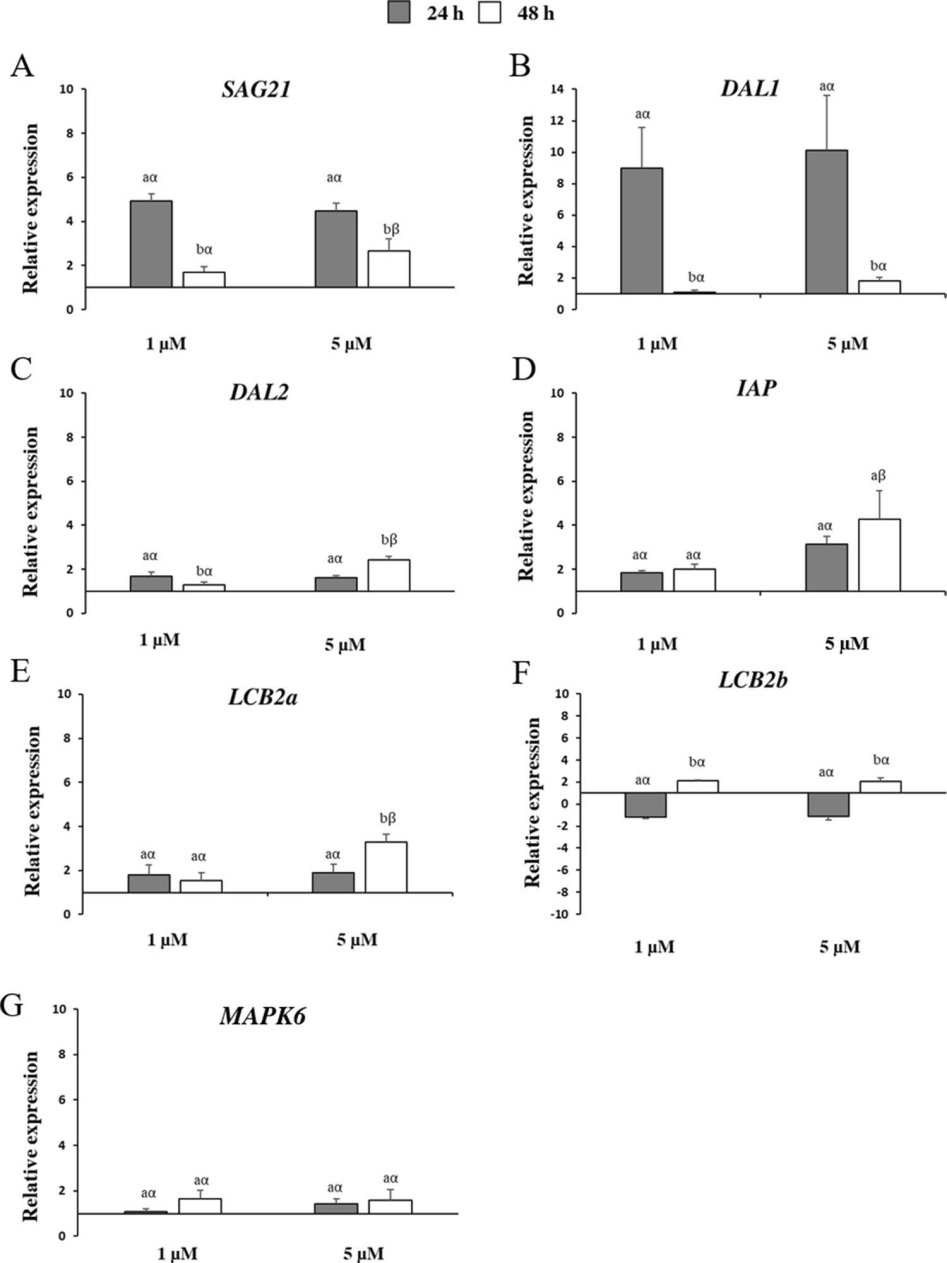
Relative expression of *Arabidopsis* genes encoding: **A** *senescence-associated gene 21* (*SAG21*); **B** *Drosophila DIAP1 like 1* (*DAL1*); **C** *Drosophila DIAP1 like 2* (*DAL2*); **D** *inhibitor of apoptosis protein* (*IAP*); **E** *long chain bases 2a* (*LCB2a*); **F** *long chain bases 2b* (*LCB2b*); **G** *mitogen-activated protein kinase 6* (*MAPK6*). Three-day-old *Arabidopsis* cells were treated with 1 µM and 5 µM of fumonisin B_1_ (FB_1_) for 24 and 48 h (grey and white bars, respectively). Vertical bars indicate ± sd. The same letters over the histograms state not significant differences between means of the two times of treatment (h) within each FB_1_ concentration (Latin letters) and the two FB_1_ concentrations within each time of treatment (h) (Greek letters), as resulting from Tukey’s honestly significant difference test (*p* < 0.05). Experiments refer to three independent biological replicates

**Fig. 4:**
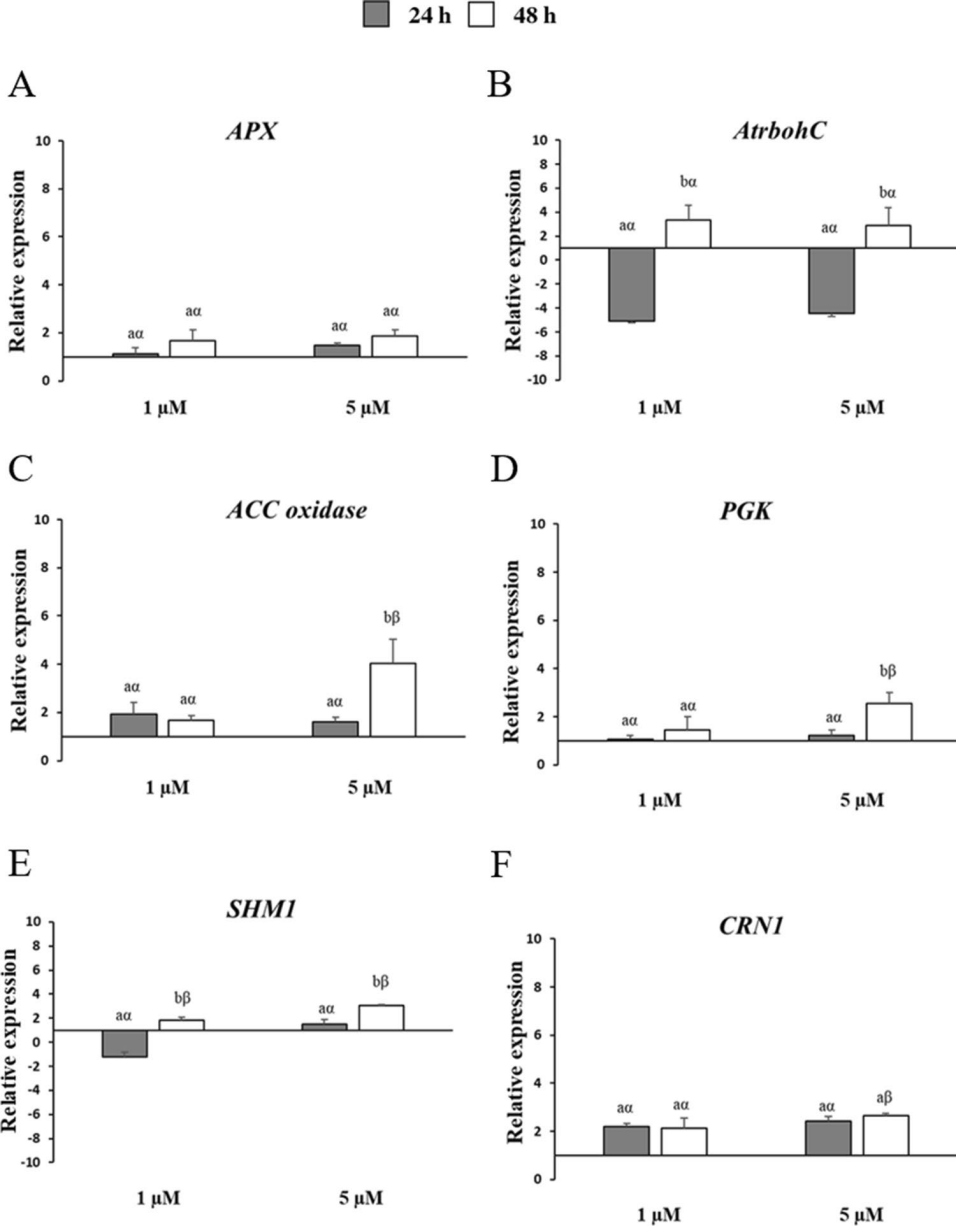
Relative expression of *Arabidopsis* genes encoding: **A** *ascorbate peroxidase* (*APX*); **B** *respiratory burst oxidase homologue C* (*AtrbohC*); **C** *aminocyclopropanecarboxylate oxidase* (*ACC*); **D** *phosphoglycerate kinase* (*PGK*); **E** *serine hydroxymethyltransferase 1* (*SHM1*); **F** *pheophytinase* (*CRN1*). Three-day-old *Arabidopsis* cells were treated with 1 µM and 5 µM of fumonisin B_1_ (FB_1_) for 24 and 48 h (grey and white bars, respectively). Vertical bars indicate ± sd. The same letters over the histograms state not significant differences between means of the two times of treatment (h) within each FB_1_ concentration (Latin letters) and the two FB_1_ concentrations within each time of treatment (h) (Greek letters), as resulting from Tukey’s honestly significant difference test (*p* < 0.05). Experiments refer to three independent biological replicates

**Fig. 5:**
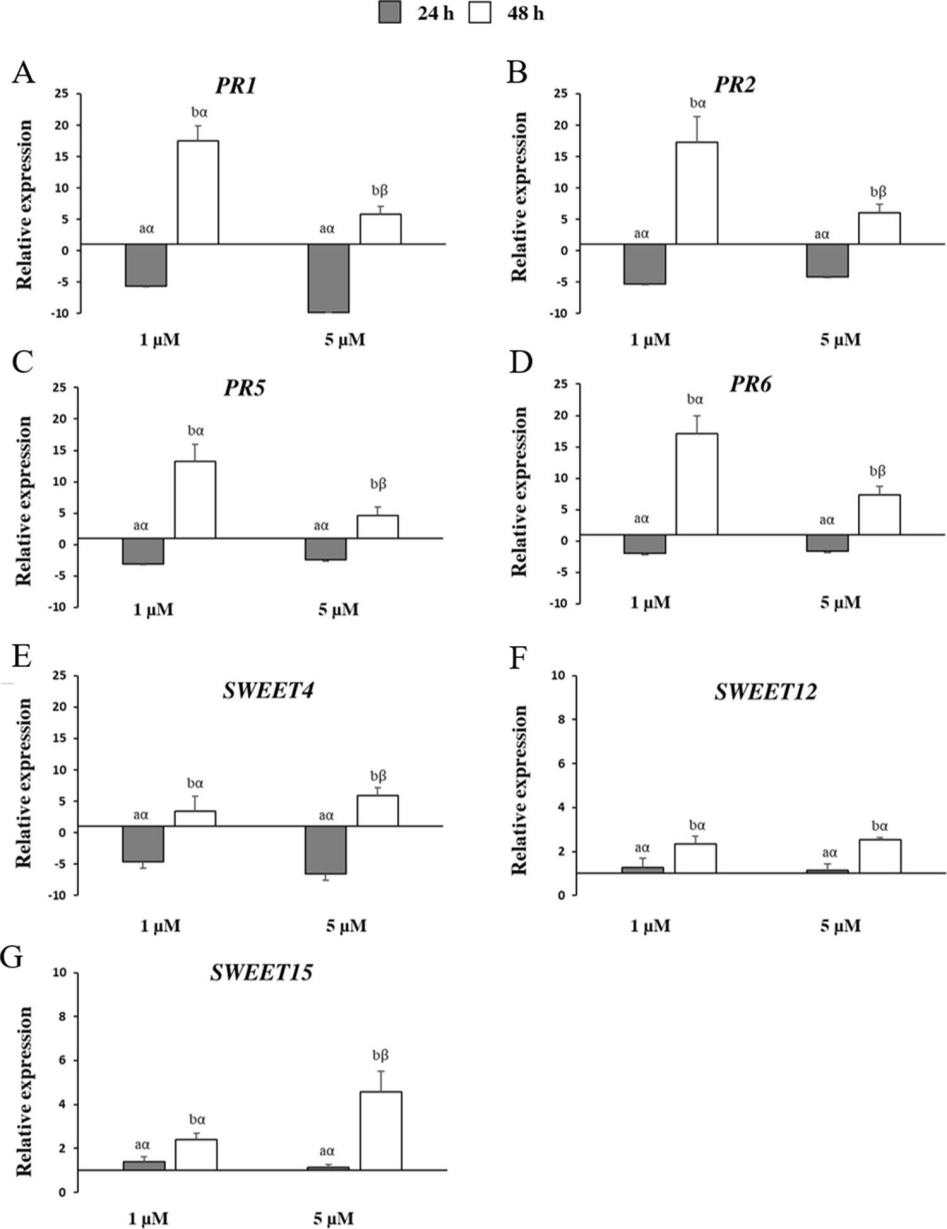
Relative expression of *Arabidopsis* genes encoding: **A** *salicylic acid-dependent defence-related* gene (*PR1*); **B** *beta-1,3-glucanase* (*PR2*); **C** *thaumatin-like protein* (*PR5*); **D** *serine protease inhibitor* (*PR6*); **E** *sugar transporter 4* (*SWEET4*); **F** *sugar transporter 12* (*SWEET12*); **G** *sugar transporter 15* (*SWEET15*). Three-day-old *Arabidopsis* cells were treated with 1 µM and 5 µM of fumonisin B_1_ (FB_1_) for 24 and 48 h (grey and white bars, respectively). Vertical bars indicate ± sd. The same letters over the histograms state not significant differences between means of the two times of treatment (h) within each FB_1_ concentration (Latin letters) and the two FB_1_ concentrations within each time of treatment (h) (Greek letters), as resulting from Tukey’s honestly significant difference test (*p* < 0.05). Experiments refer to three independent biological replicates

To verify whether a stress response took place under FB_1_ treatment, the transcriptional changes of a set of genes involved in the regulation of PCD, antioxidant metabolism, photosynthesis, pathogenesis, and sugar transport were monitored at 24 and 48 h after exposure in *Arabidopsis* cells (Figs. 3, 4, 5). Considering the previously assessed cell growth pattern by measurement of fresh weight and mortality, as well as the pattern of ROS and RNS production, these two time-points were selected as the most relevant to decipher the early molecular changes produced by the mycotoxin. Moreover, we included the 1 µM FB_1_ concentration in these analyses, to evaluate the differences between a strong and a weak dose of toxin. The relative expression profiles were calculated as fold change (FC) of FB_1_ treated over mock-treated cells.

Regarding the genes associated with the aging processes and PCD control, all assayed genes were upregulated considering both FB_1_ concentrations and times of treatment (Fig. 3). Exceptions were observed for the *long chain bases 2b* (*LCB2b*) gene at 24 h after 1 and 5 µM FB_1_ exposure (FC of − 1.2 and − 1.1, respectively; Fig. 3F). *Drosophila *DIAP1 like 1 (*DAL1*) showed the highest induction values at 24 h for both concentrations with expression levels of about 9 and 10 after 1 and 5 µM FB_1_ treatment, respectively (Fig. 3B). Similar transcriptional profiles were observed for the *senescence-associated gene 21* (*SAG21*) that significantly peaked at the same conditions (FC of about 5), followed by a decline at the later time of treatment (Fig. 3A). An opposite trend was detected for the other genes that reached a more marked upregulation almost always at 48 h after 5 µM FB_1_ treatment. This was more accentuated for the genes *DAL2*, the *inhibitor of apoptosis protein* (*IAP*) and *LCB2a* (Fig. 3C, D and E).

*SAG21* belongs to the late embryogenesis-associated (LEA) protein family and, despite being first identified as early senescence-associated gene (Hundertmark and Hincha 2008), it is also induced by H_2_O_2_ and superoxide (O_2_^.^)-donors and pathogen infection (Mowla et al. 2006; Salleh et al. 2012), thus constituting a general PCD marker. Additionally, the implication of *SAG21* in response to mycotoxin treatment in plant cells was reported in several works. Wang et al. (2012) described higher transcript levels for *SAG21* along with additional senescence-activated genes, *SAG13* and *SAG18*, and the senescence-related gene *SAG2* 8 h after ochratoxin A (OTA) treatment in *Arabidopsis* leaves. Similarly, FB_1_ exposure for a time course of 20 h stimulated *SAG21* induction in *Arabidopsis* protoplasts (Asai et al. 2012), confirming the involvement of this gene relatively shortly during PCD. *SAG21* induction contrasts with *SAG12*, which was not induced by FB_1_, nor in young control cells (Fig. 1D). The SAG12 papain-like cysteine protease is, so far, the best known senescence marker, being strongly induced in senescent leaves of *Brassica napus* L. and *A. thaliana*, especially in plants cultivated under nitrogen limitation (Desclos et al. 2008; Poret et al. 2016). Moreover, elevated SAG12 protein levels were measured in senescing leaf tissues and fallen leaves (Desclos-Théveniau et al. 2015). Nevertheless, studies carried out on *sag12* mutants did not reveal any differences in phenotypic traits and leaf senescence progression compared to wild-type plants (Otegui et al. 2005). Additionally, the lack of SAG12 was not harmful to the formation of senescence-associated vacuoles and the ribulose-1,5-bisphosphate carboxylase/oxygenase degradation (Otegui et al. 2005). Overall, SAG12 is therefore a good marker of senescence, although it is functionally not necessary to its progression. Since *SAG* genes encode for a wide family of proteases showing a broad range of sequence diversity, intracellular localizations, and expression patterns, it could be supposed that other proteases, including *SAG21*, could counterbalance the impaired expression and activity of SAG12 during senescence and other PCD events, such as FB_1_-elicited toxicity.

DAL1 and DAL2, two RING finger proteins homologous to *Drosophila* DIAP1, are functional negative regulators of PCD in *Arabidopsis*. A previous study showed that *dal1* and *dal2* mutants significantly accumulated superoxide anions, determining PCD after the inoculation of *Arabidopsis* leaves with *Pseudomonas syringae* pv. *tomato* (*Pst*) (Basnayake et al. 2011). Furthermore, the expression of *DAL1* and *DAL2* genes was abundantly increased after *Pst* and 10 µM FB_1_ treatment in wild-type plants with the highest induction at 42 h (Basnayake et al. 2011). These results are in line with those obtained from this work, since we also observed induction after FB_1_ treatments, though the peak timing and expression change intensity varied between *DAL1* and *DAL2* (Fig. 3).

Besides the DAL ring finger proteins, a further ring finger protein, the *Arabidopsis* inhibitor of apoptosis IAP showed its implication in the protection against cell death preventing caspase activation. This was pointed out by Kim et al. (2011), which reported a strong anti-apoptotic activity in transgenic *Arabidopsis* plants overexpressing *IAP* when treated with FB_1_. Furthermore, the inhibition of DNA fragmentation and caspase activity as well as an attenuated cell death caused by the bacterial effector AvrRpt2 was observed in the same plants, confirming the role of IAP as negative regulator of PCD in *Arabidopsis* (Kim et al. 2011).

Sphingolipid LCBs represent crucial PCD mediators in plants. The relationship between FB_1_ and sphingolipid pathway was previously demonstrated using *Arabidopsis* deletion mutants (Shi et al. 2007; Saucedo-Garcia et al. 2011; Kimberlin et al. 2013; Shao et al. 2019; König et al. 2021). More in detail, the insertional mutant FB_1_-resistant 11 (*Fbr11*) characterized by a deletion in the gene encoding for a LCB1 subunit of serine palmitoyltransferase (SPT) displayed lower levels of LCBs, but improved tolerance to FB_1_ (Shi et al. 2007; Kimberlin et al. 2013). Similarly, *lcb2a* mutants were unable to rise an effective PCD after 10 µM FB_1_ exposure, highlighting that the gene *LCB2a* is essential for PCD elicitation (Saucedo-Garcia et al. 2011). Furthermore, the *fbr41* mutants overexpressing the *LCB2b* gene exhibited less severe cell death phenotype when challenged with FB_1_ and *Alternaria* toxins (Shao et al. 2019). Recently, König et al. (2021) to better determine which components of the sphingolipid pool are responsible for PCD employed *fatty acid hydroxylase* (*fah1* and *fah2*) and *ceramide synthase* (*loh1*, *loh2* and *loh3*) mutants and showed that in *fah1 fah2 loh2* plants sphingolipid-induced PCD is controlled by SA signaling that in turn is influenced by the accumulation of LCBs.

LCBs are also involved in the mitogen-activated protein kinase (MAPK) cascade. Saucedo-Garcia et al. (2011) demonstrated how MAPK6 was activated in response to FB_1_ and behaved as a transducer during the LCB-induced PCD. The enhanced transcript accumulation observed in this study for the genes *LCB2a* and *b*, and *MAPK6*, predominantly at the later time of incubation (48 h) and at higher concentration of FB_1_ (5 µM; Fig. 3E–G), confirm the contribution of sphingolipid pathway to the cytotoxicity of this mycotoxin in *Arabidopsis* cells too.

The expression profiles of the antioxidant genes *ascorbate peroxidase* (*APX*) and *respiratory burst homologue C* (*AtrbohC*), the *aminocyclopropanecarboxylate* (*ACC*) *oxidase* involved in the ethylene production, the *phosphoglycerate kinase* (*PGK*), the *serine hydromethyltransferase 1* (*SHM1*) and the *pheophytinase* (*CRN1*), related to the photosynthetic and photorespiration processes, respectively, were also analyzed in this work (Fig. 4). In general, these genes showed a higher transcript accumulation during the late treatment time, more enhanced at 5 µM concentration namely for the *ACC oxidase*, *PGK* and *SHM1* (Fig. 4C–E). No significant variation was displayed by the *APX* and *CRN1* genes for both treatment times and concentrations, except *CRN1* at 48 h that resulted significantly more expressed under 5 µM FB_1_ exposure (Fig. 4A and F).

It is known that ethylene (ET) is involved in plant responses to FB_1_ and contributes to PCD and activation of defense mechanisms by a concentration and time-dependent manner (Zeng et al. 2020; Iqbal et al. 2021). Different phenotypes were observed in the *Arabidopsis* ethylene response 1-1 (*etr1-1*) mutants, probably due to the diverse light and growth conditions (Asai et al. 2012; Iqbal et al. 2021). Wu et al. (2015) by employing several ET mutants reported that sphingolipid synthesis was suppressed by ET signaling that acted as a negative regulator of FB_1_-challenged PCD. Moore et al. (1999) showed that 0.1 µM FB_1_ treatment of tomato leaflets determined an enhanced transcript accumulation of ACC synthase and ACC oxidase in the late times of exposure, in line with our findings. The increase in ACC oxidase transcript was supported by co-occurring ASC increases, the latter acting as a cofactor of the enzyme and therefore involved in the synthesis of the hormone ethylene (Smirnoff 2018). However, further research regarding the analysis of additional genes will contribute to clarify the role of this hormone in the FB_1_-induced cell death.

PCD is also induced via ROS accumulation. In this regard, it was found that FB_1_ (10 µM) elicitation rapidly induced ROS production in *Arabidopsis* leaves already after 3 days (Xing et al. 2013). Interestingly, in a further study, albeit *Arabidopsis* leaves infiltrated with FB_1_ exhibited high ROS production within 24 h, the expression of three antioxidant genes *catalase*, *APX* and *peroxidase* was not affected. In contrast, the transcript levels of *AtrbohD* and *F* slightly accumulated at 48 h in the same conditions (Qin et al. 2017). Furthermore, Wang et al. (2012) described an increased upregulation of *AtrbohC*, the same gene analyzed in this study (Fig. 4B), *AtrbohD* and *APX* after OTA treatment of excised *Arabidopsis* leaves in the first 24 h. Additional experiments focusing on different *Atrboh* isoforms and more antioxidant enzyme-coding sequences will clarify our findings more accurately in light of these previous studies.

ROS generation is greatly influenced by chloroplast metabolism and active photosynthesis (Wang et al. 2013). Stress responses against mycotoxins are often light dependent and this was earlier reported for OTA, FB_1_, and deoxynivalenol (DON) (Wang et al. 2012; Xing et al. 2013; Ansari et al. 2014). Agreeing with our outcomes, the expression of *CRN1* gene involved in the process of chlorophyll degradation was reported to be strongly induced under OTA stress (Wang et al. 2012). Conversely, *SHM1* and *PGK*, essentials for the C_2_ cycle photorespiration and carbon dioxide fixation, respectively, were suppressed (Wang et al. 2012); while in this work, they were activated of about three times at 48 h after 5 µM FB_1_ treatment. Future investigations should examine more in depth the relationship between light regulated pathways and PCD in response to the mycotoxin FB_1_.

FB_1_ also determined the induction of four pathogenesis-related genes, *PR1*, *PR2*, *PR5* and *PR6* (Fig. 5). Interestingly, the maximal transcript accumulation for all *PR* genes was measured after 48 h of treatment at 1 µM FB_1_ (average FC of about 16), whereas a downregulation was observed for both concentrations at the earlier time (Fig. 5A–D).

The elevated expression of *PR* genes upon FB_1_ exposure was previously described in several studies that tested *Arabidopsis* leaf responses. Stone et al. (2000) reported that FB_1_ elicited *PR1*, *PR2* and *PR5* induction and this trend was directly proportional to the mycotoxin concentration (0.01–1 µM). Similarly, *Arabidopsis* leaves infiltrated with 10 µM FB_1_ exhibited an elevated expression for the same genes next to *PR3* and the jasmonic acid-related *PDF1.2* response gene at 24 and 48 h, more enhanced for the late time point (Zhang et al. 2015). The accumulation of *PR1* and *PR5* transcripts was also found in the same material by Qin et al. (2017), along with ROS and salicylic acid accumulation as well as lesion formation. These two genes were strongly induced in *Arabidopsis* leaves after OTA exposure too (Wang et al. 2012). These results are partially in line with our findings, where a higher accumulation of *PR* transcripts was observed at 48 h. On the other hand, it could be assumed that the lowest transcript levels measured at 24 h are due to the different plant material examined (*Arabidopsis* leaves vs. cell cultures) and FB_1_ concentration (10 µM vs. 1 and 5 µM).

The significant role of *PR* genes was described in other species besides *Arabidopsis*, as tomato plants and maize embryos. Accordingly, the overexpression of the gene *P14a*, a member of the PR1 family, prevented FB_1_-induced PCD in tomato roots (Lincoln et al., 2018). Furthermore, FB_1_ treatment positively modulated the activity of the β-1,3-glucanase (PR2) by a concentration-dependent mode at 24 h (Sanchez-Rangel et al., 2012), emphasizing the relevance of *PR* genes as stress signaling indicators against fungal mycotoxins.

Genes encoding for sugar efflux transporters (SWEET) were also evaluated for the first time in response to FB_1_ in this study (Fig. 5E–G). The greatest expression occurred at 48 h, especially after treatment with 5 µM FB_1_, where *SWEET4* reached the most pronounced expression values (FC = 5.9; Fig. 5E). Several *SWEET* transcripts, including *SWEET4*, *12* and *15*, accumulated in response to both the bacterium *Pst* and the powdery mildew fungus *Golovinomyces cichoracearum* and *Botrytis cinerea*, highlighting the potential role of these transporters in pathogen nutrition (Chen et al. 2010a, b; Gupta 2020; Gupta et al. 2021). Previous works reported that *Arabidopsis sweet11*/*sweet12* double mutants displayed increased resistance against the fungal hemibiotroph *Colletotrichum higginsianum*, both in the biotrophic and the necrotrophic colonization phase (Gebauer et al. 2017). Additionally, *AtSWEET4* knockout mutants were found to be less susceptible to *B. cinerea* (Chong et al. 2014), suggesting that reduced carbohydrate availability correlates with susceptibility toward pathogens. Few examples in literature focusing on the role of sugar transporters in response to mycotoxins are available (Norholm et al. 2006; Vedamurthy et al. 2008; Wang et al. 2012). The expression of the hexose-specific H^+^-symporter *SPT13* was strongly enhanced in *Arabidopsis* plants challenged with FB_1_ and the virulent (DC3000) and avirulent (AvrRPM1) *P. syringae* strains 2 and 4 days after the treatment, respectively (Norholm et al. 2006). Additional sugar transporters were detected upregulated by transcriptomic analysis in response to OTA (Wang et al. 2012). A further study showed an altered glucose uptake and reduced sugar synthesis in sugarcane cells treated with the fungal red rot toxin produced by *Colletotrichum falcatum* (Vedamurthy et al. 2008).

### Antioxidant compounds and enzymes involved in the ascorbate–glutathione cycle

FB_1_ treatment was able to significantly affect the activity of the antioxidant compounds and enzymes involved in the ASC-GSH cycle, which are generally involved in the plant defense system.

**Fig. 6:**
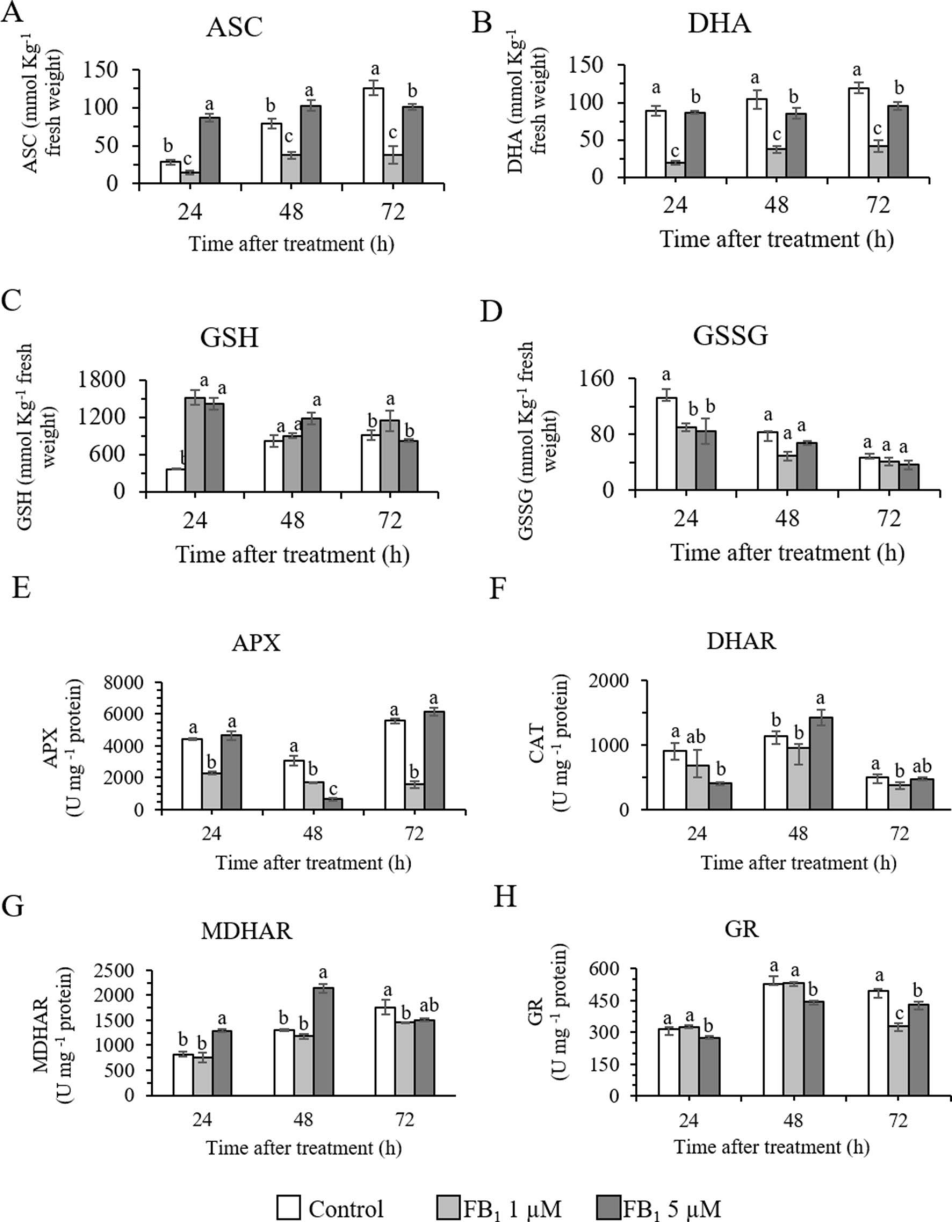
Enzymes and compounds involved in the ascorbate–glutathione cycle after fumonisin B_1_ treatment. Ascorbate -ASC (**A**), dehydroascorbate—DHA (**B**), glutathione—GSH (**C**), glutathione oxidized—GSSG (**D**), ascorbate peroxidase—APX (**E**), dehydroascorbate reductase—DHAR (**F**), monodehydroascorbate reductase—MDHAR (**G**), and glutathione reductase—GR (**H**) in control and fumonisin B_1_ (FB_1_) treated samples during 72 h of assay. One unit (U) of enzyme activity corresponds to 1 nmol of the substrate metabolized in 1 min. Letters indicate significantly different samples at each time point, according to one way Anova and Tukey’s honestly significant difference (HSD) post hoc test with *p* < 0.05. Experiments refer to three independent biological replicates

FB_1_ treatment had different responses according to the concentration applied. The lowest concentration of FB_1_, 1 µM¸ caused a marked and statistically significant decrease in ASC at all time points (Fig. 6A). This same trend was observed for DHA (Fig. 6B) and APX (Fig. 6E). As regards DHAR, MDHAR, and GR, the activity decrease was statistically significant only at 72 h (Fig. 6F, G, and H respectively), while for GSSG a decrease was observed only at 24 h (Fig. 6D). Conversely, GSH markedly increased, with statistically significant differences observed at 24 h and 72 h (Fig. 6C).

A different effect for almost all variables was observed when FB_1_ 5 µM was applied. ASC levels significantly increased at 24 h and 48 h, then were comparable to the control (Fig. 6A); in accordance to ASC trend, APX values were comparable (24 h and 72 h) or lower (48 h) with respect to the control (Fig. 6E). DHA values did not differ from the control, apart from a statistically significant decrease at 72 h (Fig. 6B). While DHAR trend fluctuated, MDHAR values remained higher than the control until 48 h and then decreased to values comparable to the control at 72 h (Fig. 6F and E, respectively).

GSH and GSSG showed opposite behaviors at 24 h, with the first being significantly higher and the latter lower with respect to the control (Fig. 6C and D, respectively). Then, a statistically significant decrease for GSH was only registered after 72 h. GR levels were always lower than the control throughout the assay (Fig. 6H).

APX is an important H_2_O_2_ scavenging enzyme, which uses ASC as electron donor in the ascorbate–glutathione (ASC-GSH) cycle. Once oxidized to MDHA, ASC is regenerated by the GSH-dependent enzyme MDHAR. DHA, originated from the disproportionation of MDHA, can be also converted to ASC by another GSH-dependent enzyme, DHAR. Finally, GSH is regenerated by GR (Loi et al. 2020a, b).

In both experimental conditions, FB_1_ affected the levels of antioxidant compounds and enzymes of the ASC-GSH cycle. When 1 µM FB_1_ was applied, the levels of the variables were generally lower, with the only exception represented by GSH. The most striking result was shown for ASC, DHA and APX, the latter being also supported by the lower levels of gene expression. These results may imply that the ASC system did not play an essential role in the H_2_O_2_ scavenging. On the other hand, we observed an increase of ascorbate at 24 and 48 h with 5 µM FB_1_ together with higher *SWEET* transcripts level, suggesting a higher availability of monosaccharides for ASC biosynthesis (Dowdle et al. 2007; Smirnoff 2018; Paciolla et al. 2019).

Conversely, GSH levels were significantly higher than the control for both experimental conditions, proving that it could be actively participating in the scavenging of H_2_O_2_ also in presence of low oxidative stress. Indeed, GSH is one of the most abundant, low-molecular-weight-thiol antioxidant molecule, involved in radical scavenging and in the protection of the thiol groups of proteins and in redox signaling (Hasanuzzaman et al. 2017). The increase in GSH cannot be ascribed to an increase of GR, neither to the activity of MDHAR and DHAR. It is therefore possible that other enzymes contributed to maintain high GSH levels when FB_1_ was applied. GSH homeostasis is redundantly regulated at different levels, which control the synthesis, the degradation, and the regeneration from its oxidized form (Hasanuzzaman et al. 2017). Moreover, ER stress is reported to increase GSH levels in *Arabidopsis*, possibly due to the downregulation of GSH-dependent peroxidases (Uzilday et al. 2017).

### Enzymes involved in H_2_O_2_ scavenging and H_2_O_2_ levels

Different enzymes involved in H_2_O_2_ scavenging, namely SOD, POD, and CAT, were considered in this study to assess the effect of FB_1_ on the oxidative response of *Arabidopsis* cells.

**Fig. 7:**
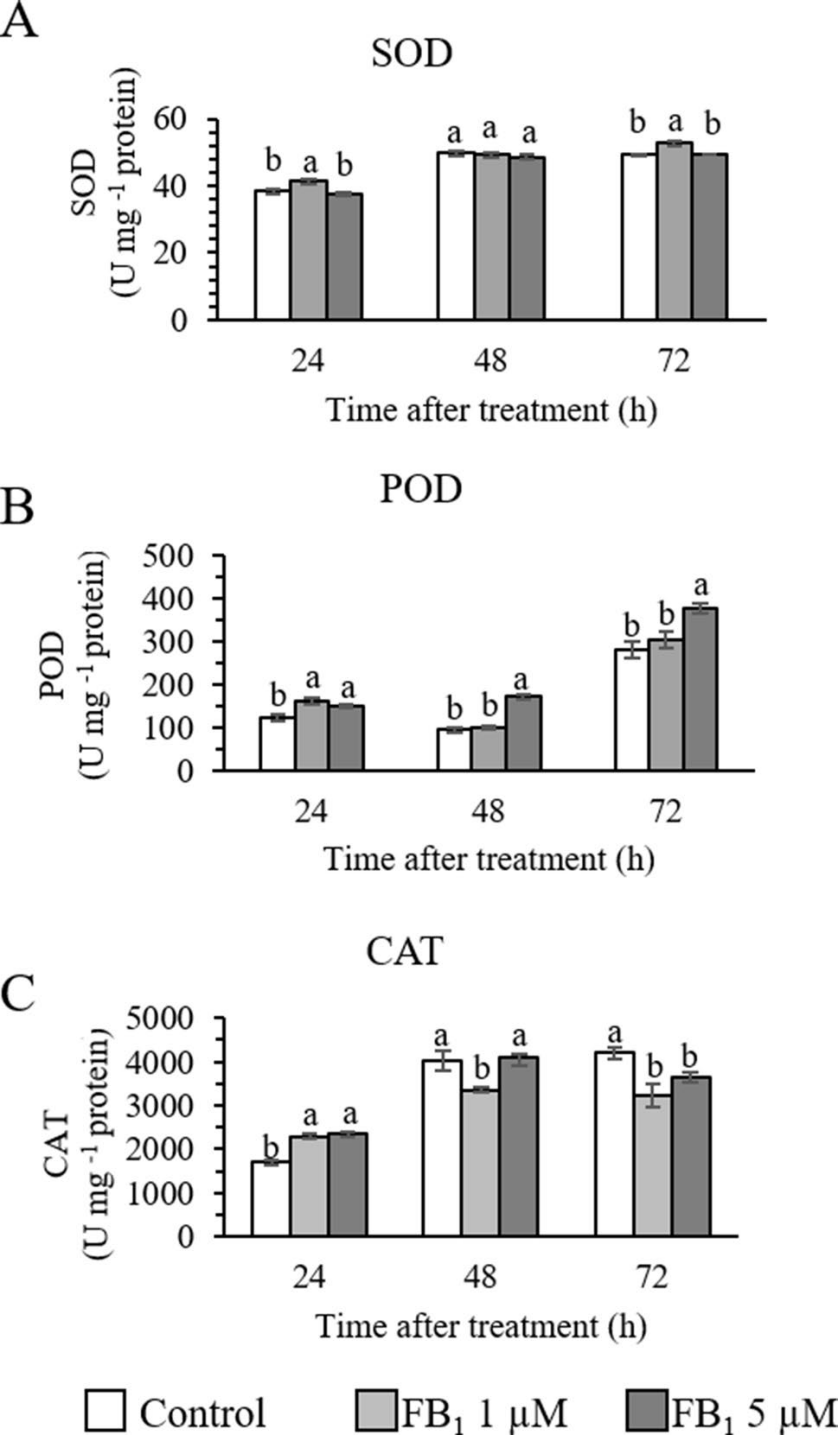
Enzyme activity of superoxide dismutase—SOD (**A**), peroxidases—POD (**B**), and catalase—CAT (**C**) in control and fumonisin B_1_ (FB_1_) treated samples during 72 h of assay. One unit (U) corresponds to 1 nmol of the substrate metabolized in 1 min. Letters indicate significantly different samples at each time point, according to one way Anova and Tukey’s honestly significant difference (HSD) post hoc test with *p* < 0.05. Experiments refer to three independent biological replicates

FB_1_ (1 µM) induced a slight, but statistically significant increase in SOD after 24 h and 72 h (Fig. 7A), and in POD, though only after 24 h (Fig. 7B). CAT levels were also increased by 1 µM FB_1_ at 24 h; nonetheless, at 48 h and 72 h they were lower than to the control (Fig. 7C). The same trend was elicited by 5 µM FB_1_ for POD and CAT (Fig. 7B and C), while no differences with the control emerged for SOD (Fig. 7A).

H_2_O_2_ is one of the most important ROS, endowed with a relatively long half-life and high diffusion rate in water (Smirnoff and Arnaud 2019). Due to those characteristics, at low concentrations H_2_O_2_ acts as a signal molecule, regulating the redox balance of the cell, its growth and development. Several enzymatic and non-enzymatic compounds are redundantly involved in ROS and H_2_O_2_ scavenging to assure that a physiological level is maintained. In addition, specific LCB can induce early ROS production and cell death, requiring respiratory burst oxidases (Peer et al. 2011).

In our experimental condition, FB_1_ was able to induce a rapid increase in intracellular H_2_O_2_ throughout the assay, causing reduced cell growth and, eventually, cell death.

Based on our data, we hypothesize that during the first hours of exposure, intracellular H_2_O_2_ was scavenged due to an increase in CAT and POD activity, although the SOD activity increased at 24 h with 1 µM FB_1_, contributing to H_2_O_2_ increase; this, however, together to parallel increase of *DAL1* and *DAL2* gene transcripts kept under control the radical superoxide anion level (Basnayake et al. 2011). Later on, the system entered in physiological distress, H_2_O_2_ kept accumulating without being counteracted by CAT and POD, contributing to cell death.

Following these findings, the discrepancy in intracellular H_2_O_2_ with the results obtained for the extracellular H_2_O_2_ (which was higher with 5 µM FB_1_) can be explained by a leakage of H_2_O_2_ from the cellular compartment to the extracellular environment. Besides, H_2_O_2_ can be produced by separate systems in the plasma membranes and cell walls, such as the NADPH-dependent oxidases which are implied in the cell wall H_2_O_2_ –dependent lignification (Habibi 2014).

### Isozymes and protein redox status

The isozyme pattern was analyzed by native-PAGE. No differences emerged between the control and the samples treated with FB_1_, regardless of the concentration used (data not shown). Therefore, *Arabidopsis* response to FB_1_ did not involve the induction of additional isozymes for all enzymes analyzed (APX, CAT, GR, SOD, DHAR, and POD). So far, the induction of novel isozymes with DHAR activity and involved in the defense mechanism has been reported in tomato plants for beauvericin, another *Fusarium* toxin (Loi et al. 2020b).

The redox state of protein-thiols appeared unchanged (data not shown), with no differences between the control and the FB_1_ treated samples, possibly maintained by the high GSH levels through glutathiolation, a reaction that can protect the protein thiol groups from irreversible inactivation by oxidation (Rouhier et al. 2008; Rouhier et al. 2015). The glutathiolation, that is a reversible post-translational modification consisting in a disulfide bond formation between a protein thiol and GSH, occurs more frequently in response to increased ROS (Rouhier et al. 2008).

## Conclusions

The data set from this study offers significant insight into concentration and time-dependent responses of *Arabidopsis* cell culture to FB_1_. FB_1_ exposure to *Arabidopsis* cell culture induced a stress response leading to cell death, which might be due to a strong oxidative and nitrosative damage. Cell death showed hallmarks typical of rapid HR response, as opposed to a slow senescence-like program. The early production of RNS was followed by a later ROS burst, possibly indicating a general mechanism for PCD induction in plant cells. The transcriptional analysis revealed that FB_1_ was able to induce different genes involved in the regulation of PCD, antioxidant metabolism, photosynthesis, pathogenesis, and sugar transport. Among the biochemical parameters studied, GSH seemed to be the main antioxidant compound involved in the stress response to the fumonisin exposure, highlighting the pivotal role of this multifaceted antioxidant molecule.

Collectively, the outcomes of this work showed that FB_1_ exposure probably induced several redundant defense networks in *Arabidopsis* cells, pointing out the complex and dynamic plant-toxin interaction. Although further studies are needed to completely elucidate this multifaceted signaling network, the results of this work describe the general response of cultured *Arabidopsis* cells to FB_1_ exposure at the physiological, molecular and biochemical levels.

## Supplementary Information

Below is the link to the electronic supplementary material.

Supplementary file1 (DOCX 14 KB)
