# Supplementary material for: Cell death induced by mycotoxin fumonisin B1 is accompanied by oxidative stress and transcriptional modulation in Arabidopsis cell culture
Source: Plant Cell Rep. 2022 Jun 25;41(8):1733–50. doi: 10.1007/s00299-022-02888-5 (PMC9304057; doi:10.1007/s00299-022-02888-5)
Supplement: Supplementary file 1 — Supplementary file1 (DOCX 14 KB) [file 299_2022_2888_MOESM1_ESM.docx]

| Gene | Primer Forward (5’🡪3’) | Primer Reverse (5’🡪3’) | Source |
| --- | --- | --- | --- |
| *Actin2* | GAATCGCCGACAGAATGA | TACTGAGGGAGGCCAAGA | Wang et al., 2012 |
| *SAG21* | GGAAGAAGTGGAGCTGTTGC | CCGGTTTCGGGTCTGTAATA | This study |
| *DAL1* | GTTCCTTGGTTTCTGGACGA | CAGTTGGAAGAACACGCTCA | This study |
| *DAL2* | TTGTTGATGCAGCTGCTAGG | TAGTTGCAAGGAGCATGGTG | This study |
| *IAP* | GGCTGTTCAAGCTCATCACA | CTCAACGGCGAAAGATTCTC | This study |
| *MPK6* | TCCATCAAATCATTCGGTCA | AGTTTGCGTTCAGGAGGAGA | This study |
| *LCB2a* | GCCGGCGATAATAAACTCAA | CCACCACCACCACTAGGTCT | This study |
| *LCB2b* | TTCCCGTCTTGATTGGAAAG | GTCTATGCGTCCTGGGTTGT | This study |
| *APX* | GCACCAGGAGGACAGTCATG | GGGCTACAGCGTAATCCTTG | Wang et al., 2012 |
| *AtrbohC* | TCACCAGAGACTGGCACAATAAA | GATGCTCGACCTGAATGCTC | Wang et al., 2012 |
| *ACC oxidase* | AGGAAACAGGATGTCGGTTG | CTGAAACTTGACCCCTGCAT | This study |
| *PGK* | TCTCCACTCATCTGGGAAGG | CATCAGCTTTCGTGACCTCA | Wang et al., 2012 |
| *SHM1* | GTTGCTGGTGCAAGTGCTTA | GGGTCCACGAAGTGACTTGT | Wang et al., 2012 |
| *CRN1* | GGTGAGACCGTTATGGGGAAAG | GCATCAGATAGTTCACCACCTCAG | Wang et al., 2012 |
| *PR1* | AAGGAGCATCATATGCAGGA | ATTTAAATAGATTCTCGTAATCTCAGC | Wang et al., 2012 |
| *PR2* | GCAATGCAGAACATCGAGAA | CATCCCTGAACCTTCCTTGA | This study |
| *PR5* | GATGGCGGCAAAGATTTCTA | GCGTTGAGGTCAGAGACACA | This study |
| *PR6* | AGAGAACTCGAGCCTCAACG | CGATACGGATTTCGTCAACC | This study |
| *SWEET4* | CCATCATGAGTAAGGTGATCAAGA | CAAAATGAAAAGGTCGAACTTAATAAGTA | This study |
| *SWEET12* | AAAGCTGATATCTTTCTTACTACTTCGAA | CTTACAAATCCTATAGAACGTTGGCAC | This study |
| *SWEET15* | CAATGACATATGCATAGCGATTCCAA | GGACTCATCACGACAATACTCTTAAG | This study |

**Supplemental Table S1** Primer sequences for real-time RT-PCR analyses
